# Physiological Indicators of Attachment in Domestic Dogs (*Canis familiaris*) and Their Owners in the Strange Situation Test

**DOI:** 10.3389/fnbeh.2019.00162

**Published:** 2019-07-23

**Authors:** Morag G. Ryan, Anne E. Storey, Rita E. Anderson, Carolyn J. Walsh

**Affiliations:** ^1^Cognitive and Behavioural Ecology Program, Memorial University of Newfoundland, St. John’s, NL, Canada; ^2^Department of Psychology, Memorial University of Newfoundland, St. John’s, NL, Canada; ^3^Department of Biology, Memorial University of Newfoundland, St. John’s, NL, Canada

**Keywords:** dog, attachment, cortisol, chromogranin A (CgA), strange situation test

## Abstract

Behaviorally, attachment is demonstrated when one individual maintains close proximity to another individual and shows distress upon separation. For 29 owner-dog dyads, we employed a modified Ainsworth’s Strange Situation Test (SST) to investigate whether both members would show a physiological reaction to separation. Dogs experienced a series of separation from and reuniting events with their owners and were introduced to a stranger. Before and after the SST, saliva samples were taken from each dyad to measure stress-related analytes: cortisol (CORT) and chromogranin A (CgA). Dogs exhibited attachment behaviors toward owners as evidenced by more time spent in close proximity, more contact initiated and less time spent near the door, compared to episodes with the stranger. Dogs that initiated more contact with their owners in re-uniting episodes had lower CgA than dogs that initiated less contact, but their owners had higher CgA levels. Also during re-uniting episodes, dogs and owners spent more time near each other when owner CgA levels were low, owner CORT levels were high, and the dog had owner-reported separation anxiety. During the episodes alone with the stranger, dogs with higher CORT spent more time with the stranger. Finally, dogs’ initial CgA levels were correlated with their owner’s initial CORT levels, and dog final CORT levels were correlated with their owners’ final CORT levels, suggesting some hormonal synchrony within the dyad. As all owner-dog dyads were assessed as securely attached, attachment style differences could not explain variation in hormonal or behavioral results. These results suggest that dogs may respond to owner hormonal state and/or behavior and demonstrate that individual differences in responses to a behavioral challenge reflect the stress physiology of both dogs and their owners.

## Introduction

Behaviorally, attachment is defined as one individual seeking and maintaining close proximity to another individual and exhibiting distress upon separation (Bowlby, 1958, 1972; Ainsworth, 1969; Klagsbrun and Bowlby, 1976). The “attachment figure” is often used as a “secure base” for exploration, providing social and emotional support required for handling stressful situations and new environments (Ainsworth, 1979, 1989; Waters and Cummings, 2000; Mikulincer et al., 2014). Consequently, individuals show a distinct preference for their attachment figure(s) and typically experience a stress response upon separation (Insel and Young, 2001), perhaps even an exaggerated stress response, as seen in individuals with “separation anxiety” (Voith and Borchelt, 1985; McCrave, 1991; Appleby and Pluijmakers, 2004; Ogata, 2016).

We know much less about cross-species attachment than we do about the better-studied parent-child relationship. Given our long history of cohabitation, the dog-human bond is ideal for determining whether the behavior and physiology of attachment transcend human bonds.

The relationship between humans and domesticated dogs (*Canis familiaris*) has undergone thousands of years of shared evolutionary history, likely tapping into similar neurobiological substrates for attachment (Serpell, 2003; Miklósi, 2007; Nagasawa et al., 2009; Mongillo et al., 2010; Axelsson et al., 2013; Topál et al., 2014). This is not to say that this relationship is entirely analogous to a parental model. However, Companion dogs, in particular, rely on owners for basic needs and many have been created, through artificial selection, to retain infantile features (e.g., large eyes, bulging cheeks) throughout adulthood (Coppinger et al., 1987; Archer and Monton, 2011). Therefore, it is not surprising that domesticated dogs are able to elicit human care-giving responses.

It is evident that humans form attachments to their dogs and in some cases preferentially seek comfort from them rather than another person (Kurdek, 2009). The strength of owner attachment is related to the dog’s behavior, as owners who are “satisfied” with their pet’s behavior are generally more strongly attached (Serpell, 1996). Humans also derive many health and social benefits from dog ownership. Links have been found between dog ownership and lower blood pressure and heart rate, increased physical activity and higher survival rates. These links suggest that the social support and exercise provided by dog companions aids in buffering against negative stressors (Friedmann et al., 1983; Garrity et al., 1989; Serpell, 1991; McNicholas et al., 2005; Kurdek, 2009; Bushman, 2014; Krause-Parello et al., 2014). The idea of a buffering role is supported at a short-term physiological level, as dog owners experience decreases in blood cortisol (CORT, a stress hormone) levels when making physical contact with their dogs (Handlin et al., 2011). Dogs also play an important role in therapeutic situations, allowing both children and adults to express emotions related to attachment, sometimes more easily than they can to other people (Zilcha-Mano et al., 2011; Julius et al., 2013).

Similar to humans, dogs exhibit behavioral manifestations of attachment by showing a preference for owners over strangers. Compared to non-owners, dogs spend more time in close proximity, pay more attention, i.e., gaze duration (e.g., Mongillo et al., 2010; Horn et al., 2013; Kerepesi et al., 2015), and react more to the absence of their owners (e.g., Tuber et al., 1996; Konok et al., 2011; Mariti et al., 2013). Researchers have investigated human-dog attachment using Ainsworth’s Strange Situation Test [SST; 3], a procedure for assessing attachment styles of young children to their parents [i.e., secure or insecure; (Ainsworth, 1979)]. This behavioral protocol subjects a dependant to a series of separation and reuniting events from her/his attachment figure and introduces dependants to a stranger to determine responses to separation and reunion, and strength of the attachment relationship (Tuber et al., 1996; Topál et al., 1998, 2005; Gácsi et al., 2001; Prato-Previde et al., 2003; Palestrini et al., 2005; Fallani et al., 2007; Palmer and Custance, 2008; Konok et al., 2011; Mariti et al., 2013; Mongillo et al., 2013; Rehn et al., 2013, 2014; Schöberl et al., 2015). Although most canine modifications of the SST involve quantifying dog and owner behaviors, the qualitative infant-parent attachment classifications (e.g., secure vs. insecure) also have been used to describe this relationship in several studies (e.g., Schöberl et al., 2015; Solomon et al., 2018; Wanser and Udell, 2019).

As addressed, a mainstay of attachment is stress upon separation, which is why it is important to discuss the relative neuroendocrine systems that control the stress response. Organisms show both “fast” and “slow” physiological responses to stress. The slower response system is the hypothalamic-pituitary-adrenal (HPA) axis, which controls the secretion of cortisol (CORT), a steroid hormone produced and released by the adrenal cortex in response to psychosomatic and physical stress (Kudielka et al., 2009; Frodi and O’Keane, 2013; de Veld et al., 2014). CORT has been successfully measured in saliva in both dogs (e.g., Beerda et al., 1998; Bergamasco et al., 2010; Ottenheimer Carrier et al., 2013; Schöberl et al., 2015) and humans (e.g., Hellhammer et al., 2009; Kudielka et al., 2009). Salivary CORT levels have also been found to correlate strongly with levels found in plasma, albeit at lower concentrations (e.g., Lebelt et al., 1996; Calixto et al., 2002).

A measure of the faster physiological stress response system, the sympathetic adrenomedullarly system (SAM), is an acidic protein called chromogranin A (CgA). CgA is a novel stress marker, which is co-released with catecholamines (epinephrine and norepinephrine) from the adrenal medulla and sympathetic axons (van Kammen et al., 1992; Kanno et al., 1999; Stefanescu et al., 2011). CgA is an excellent tool for measuring SAM activity because it is more stable than catecholamines in the circulatory system, as it lasts longer and is consequently easier to measure, especially in saliva (Kanno et al., 1999). Like CORT, CgA concentrations in the saliva have been measured in both dogs (Akiyoshi et al., 2005; Kanai et al., 2008) and humans, and salivary and plasma levels are highly correlated (Nakane et al., 1998, 2002; Den et al., 2011; Stefanescu et al., 2011). Using CORT and CgA should allow a more precise determination of both slower and faster physiological changes, respectively (or, potentially, levels of perceived stress severity).

This study investigated the physiological responses of dogs and their owners to the Strange Situation Test (SST). In keeping with previous studies of dogs tested in the SST, dogs should show clear behavioral indicators of attachment to their owners by maintaining proximity to, initiating more contact with, and showing increases in stress-related behaviors (e.g., door scratching) when separated from them, as compared to strangers. We predicted the separation and reuniting events would elicit HPA and/or SAM activity, resulting in CORT and CgA increases (final > initial levels), and relationships between dogs’ physiological measures and attachment behaviors they exhibited during the SST. Since these measurements could be influenced by owner-reported separation anxiety (SA), we tested whether SA affected our results. Finally, because of the relationship between dogs and their owners, we predicted that physiological indicators of separation-induced stress would also be observed in owners, an aspect of the dog-owner relationship that has not previously been studied.

## Materials and Methods

### Ethics Statement

This study was carried out in accordance with Canada’s Tri-Council Policy Statement: Ethical Conduct for Research Involving Humans (T 2) and regulations of the Canadian Council on Animal Care (CCAC). Permits for this specific research project were issued under the auspices of Memorial University of Newfoundland’s Interdisciplinary Committee on Ethics in Human Research (ICEHR #2012-320-SC) and Institutional Animal Care Committee (IACC protocol #12-01-CW). As per approved protocol in ethics permit ICEHR #2012-320-SC, written consent was obtained from participants (owners) prior to their participation in the study.

### Participants

We tested 29 volunteer owner-dog dyads. Three additional dyads began testing but their participation was ended when the dogs became extremely apprehensive in the experimental situation (e.g., panting excessively, trembling, etc.). Owners were given a complimentary dog bag dispenser at the end of the study, but were unaware that they would receive this gift prior to participation. In an attempt to obtain a representative sample of Newfoundland dog owners, participants were recruited through a variety of social media (e.g., public posters, booths at dog shows and at a local Pet Expo, departmental e-mails and local classified ads such as www.kijiji.ca). Owners consisted of six men and 23 women, ranging from 20 to 71 years old (mean age ± *SD* = 40 ± 14.8 years). There were 13 male and 16 female dogs, ranging from 8 months to 14 years old (mean age ± *SD* = 6 ± 3.9 years).

Of the 29 dogs tested, five were sexually intact: one female (not in estrus at the time of the study) and four males; the remaining 24 dogs were neutered/spayed. A variety of medium to large (∼9–40 kg; Supplementary Table S1) dogs were used (with the exception of one Yorkshire Terrier) to ensure that enough saliva could be obtained. All dogs were kept strictly for companionship or recreation purposes, i.e., there were no working or service dogs in this study. The majority of households (*N* = 19, 66.0%) had one dog at the time of the study; the remainder of households owned multiple dogs (maximum of four dogs).

Prior to participation, dogs and owners were screened to ensure that they were free from endocrine pathologies that could influence adrenal gland function and that dyads had cohabitated for at least 6 months. None of the dogs had aggressive tendencies and all were familiar with traveling outside of their homes. Of the 29 dyads tested, 28% (*N* = 8) of owners reported that they believed their dog had separation anxiety (SA), or that they had been told by a veterinarian or trainer that their dog had separation anxiety. As the presence of separation anxiety could influence SST behaviors and stress analytes, this factor was accounted for in all statistical analyses.

Recruitment occurred between August 9, 2012 and February 25, 2013. Owners and dogs were tested at Memorial University of Newfoundland between 1300 and 1900 h, to minimize the effects of circadian fluctuations [e.g., CORT is highest in the morning; (Wüst et al., 2000)]. Participants were asked to refrain from: eating (especially dairy products) 1 h before arrival, drinking caffeine 2 h before arrival and excessively petting their dogs on route to the study location, as these factors may influence salivary results (Hofman, 2001; Kaufman and Lamster, 2002; Handlin et al., 2011; Schultheiss et al., 2012).

Following testing of the first three owner-dog dyads, the location of the testing room changed. Since, we were concerned about the potential effect of test location change on the dogs’ behaviors, their data were excluded from the final analyses (final *N* = 26, including all 8 dogs with owner-reported SA).

### Study Location

**FIGURE 1:**
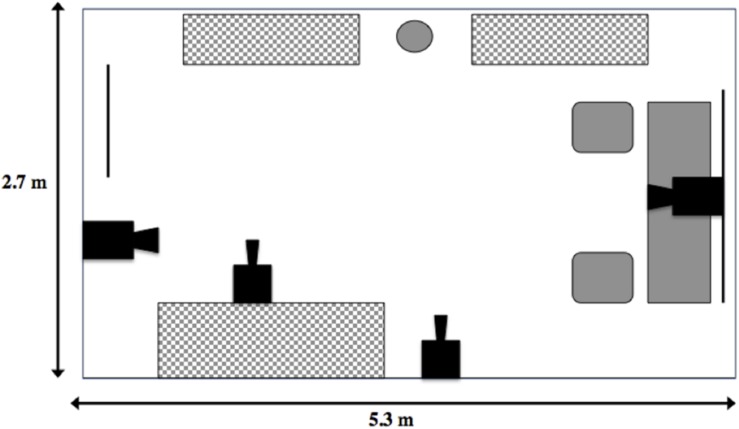
Layout of the Strange Situation room. Solid black objects represent camera placements in the room, the circle represents the water dish available to the dog, the gray and white checker rectangles represent storage units, and the solid gray shapes represent the desk area where the sample supplies, speakerphone and basket of toys were stored. Solid black lines represent a window (immediate right) and door (immediate left).

The main study location was a 2.7 m × 5.3 m office, located in the Science Building at Memorial University, which contained a desk, two chairs, a speakerphone, a basket of toys, a water bowl and a series of storage units (filing cabinets and book shelves; Figure 1). The toy basket contained 7 toys (e.g., squeaky Kong^®^ rings, medium black rubber Kong^®^ chew toys), which were washed between tests. Four synchronous security cameras (LH114000 series, Lorex, Plainsfield, IL, United States) were set up in the room at a variety of angles, so that most of the room was captured on video. The cameras were connected to a hard drive (where the recording was stored) and a monitor. Office cooling fans were also placed in the room to mask ambient noise (e.g., experimenter and owner talking in the corridor), to maintain a comfortable room temperature (∼20°C) and to keep trials as similar as possible.

### Strange Situation Procedure

The Ainsworth Situation test (Ainsworth, 1969) was amended for dogs as in previous research (e.g., Prato-Previde et al., 2003; Palestrini et al., 2005; Palmer and Custance, 2008; Rehn et al., 2014). The basic protocol involves a dog experiencing a series of separation and reuniting events from her/his owner and exposure to a stranger. Five different strangers were used in the study; each dog experienced a single stranger. One stranger (MC) tested 20 dogs, two strangers tested two dogs each, and two strangers tested one dog each. It was necessary to use multiple strangers, as some dogs were familiar with some assistants, and we wanted to ensure that no dog had previous interactions with the stranger used during their test. All strangers were females between the ages of 20–30 years old with prior experience interacting with and obtaining saliva samples from unfamiliar dogs. Strangers received standardized instructions on how to behave toward the owners and dogs, such that all strangers performed their roles in a highly similar manner. Comparison of the hormones and measured behaviors in dogs (*N* = 20) for whom MC acted as stranger to the remaining dogs (*N* = 6) revealed no significant differences.

**FIGURE 2:**
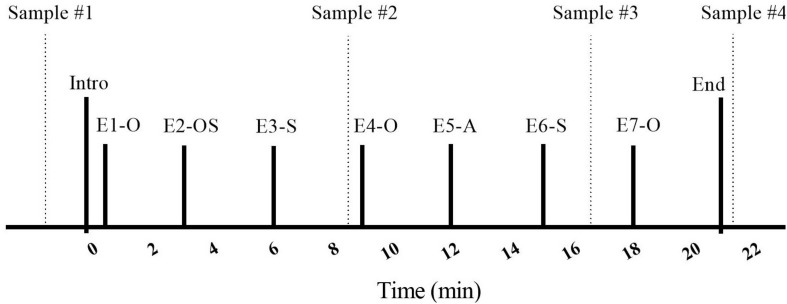
Timeline of the Strange Situation Test (SST) protocol. E, Episode; O, Owner; S, Stranger; A, alone; dashed vertical lines indicate saliva sample taken.

The procedure involved the researcher briefly introducing the owner and dog to the room (30 s), followed by seven “episodes,” each lasting 3 min (Figure 2). Ten minutes after the dyads arrived, and prior to entering the SST room, initial saliva samples were taken from both owners and their dogs by the primary researcher (MR). The 10-min delay was used to ensure that the dyad had some time to adjust to their arrival at the testing location. Owners and dogs were then introduced to the room and contents. Owners were told that they should interact with their dogs in whatever manner they pleased, and that they could use the toys from the basket if they wished. Specific instructions on how to play with their dogs were not given, as we wished to have the owner-dog interaction reflect the natural style of each dyad. Instructions for episode transitions and for the timing of saliva sampling were given by the experimenter over a speakerphone located on the desk. This was followed by a series of separation and reuniting episodes in which the dog was with the stranger, the owner, with both, or was left alone.

Each dog and owner entered the room for the Strange Situation Test (SST) at the beginning of the Introduction, which was followed by seven 3 min episodes. Episodes involved the entrance/exit of a stranger, separation from and reuniting with the owner, and the dog being left alone. After each episode title, letters represent the individual(s) present in the room with the dog (O: owner, S: stranger), or reflect that the dog was alone (A: alone). Following the initial (baseline) saliva sample, taken before the SST procedure, saliva samples were taken at the times indicated by vertical lines during Episodes 3, 6, and 7.

### Saliva Sampling

Two sampling techniques were used to collect saliva: a swab technique for dogs and the passive drool technique for humans. For dogs, the individual taking the sample held an 8 mm × 125 mm swab (Salimetrics Children’s Swab, © Salimetrics, PA, United States) made from a durable, inert polymer in the dog’s mouth (typically near the cheek) until saturated (<1.5 min). The swab was then placed within a 17 mm × 100 mm polypropylene, barcoded tube (Swab Storage Tube, © Salimetrics, PA, United States) and laid on ice. Owners were asked to lean their head forward, allow the saliva to pool in their mouth and then to guide that saliva into a 10 mm × 46 mm polypropylene tube (Passive Drool Cryovial, © Salimetrics, PA, United States) using a collection device (Saliva Collection Aid, © Salimetrics, PA, United States) similar to a straw, and then the tube was placed inside the ice chest. Collection supplies were chilled on ice prior to use. Only the initial and final saliva samples for dogs and humans were analyzed and reported, mainly because many intermediate samples for dogs did not have adequate saliva for analyses. It was also felt that the initial and final values would be the most meaningful due to the known time course of salivary CORT in response to a stressor [e.g., peaking at ∼20 min and returning to near baseline by 40 min, (Dreschel and Granger, 2005)], whereas the CgA time course was still relatively unstudied, which is still the case for dogs. However, more recently published work in pigs indicates that CgA is a reliable indicator of stress induced by restraint [increasing ∼10 min following restraint (Huang et al., 2017)].

### Salivary Analyses

Immediately after testing, samples were placed in storage containers in a −20°C freezer until they were shipped, immersed in dry ice, to Salimetrics LLC (State College, PA, United States). Each sample with adequate volume was analyzed in duplicate for two stress markers: CORT and CgA. Both analytes were measured using Enzyme Immunoassay (EIA); Cortisol, 1-3002 (Salimetrics, State College, PA, United States) and Chromogranin A, YII-YK070-EX (Cosmo Bio CO., LTD., Japan), respectively. Concentration values were expressed as μg/dL for CORT and pmol/mL for CgA. Sometimes there was not enough volume of saliva to run both tests. In those cases, CORT was the highest priority and it was always measured. We chose CORT as the priority analyte because more previous research existed for comparison (e.g., Mongillo et al., 2013; Schöberl et al., 2015). This priority reduced the number of CgA values available for dogs (dog initial CgA, *N* = 18; final CgA, *N* = 20; both initial and final CgA, *N* = 15).

### Behavior

Videos converted to .mp4 files were watched using QuickTime Media Player 7 (Apple, Toronto, ON, Canada), synchronized with a behavioral coding program logger.app^1^ (©Avery Earle, Memorial University of Newfoundland). This coding program synchronized with the video’s time signature and allowed a one-letter code to be assigned to each behavior, providing a time stamp for the behavior. The resulting .txt data files were processed using a unique Python code (programming language) on an Apple interface to extract durations and frequencies of the behaviors analyzed.

The behaviors coded included those likely to indicate two aspects of attachment: seeking proximity to and showing distress when separated from the attachment figure (owner). Thus, proximity of the dog to the owner and, for comparative purposes, to the stranger, as well as physical contact initiated by the dog were recorded. Proximity to the door was measured as both a possible indicator of distress (e.g., the dog is in a novel room, and the door is a means of exiting), and of seeking proximity to the owner during episodes when the owner is out of the room. Door scratching by the dog is similarly assumed to reflect distress and/or proximity-seeking toward owner. Shake off was measured as a possible indicator of stress or coping (Glenk et al., 2013).

Dogs were considered to be in close proximity to a person or object if they were within one distance of their own body length (snout to rear) from a person(s) or object(s). Proximity of the dog to a human (owner or stranger), and to the door, was coded for all episodes and was analyzed for each episode separately. All proximity durations were analyzed as a proportion of total time the focal individual had available to interact with the dog (i.e., the time required for the stranger to take saliva samples was removed from the total time used to calculate “dog-stranger proximity” and “door proximity” for Episodes 3 and 6). The duration and frequency of physical contact between dogs and humans were coded when the dog was in proximity to the owner or stranger for each episode. Rates of dog- vs. owner-, or stranger-initiated contact were coded separately; to be coded as dog-initiated contact, clear indication of a movement goal (forward gaze, approaching the human) was required. Shake off was defined as any one continuous bout of side-to-side movement starting at the head and extending down the body (as if the dog was drying off). Door scratching was counted each time the dog made contact with the door using a paw. A new bout was counted when contact was broken (i.e., all paws on the floor) and then resumed. Table 1 lists all behaviors analyzed.

**TABLE 1:** Ethogram of behaviors recorded and measured.

**Behavior**	**Definition**
Physical proximity to Owner, Stranger, and Door	Physical closeness to focal individual/object within the distance of the dog’s own body length (snout to rear); measured as duration.
Physical contact with Owner and Stranger	Contact occurring between a person and the dog, including human-initiated behaviors such as touching, stroking, patting, and extended touch (making physical contact using a toy or touching/pulling the dog’s collar), as well as dog-initiated behaviors such as jumping up on, sitting on, nosing, and pawing; measured as rate (frequency/second). Physical contact during saliva sampling was excluded.
Shake off	A side-to-side motion that begins at the head and extends down the body. This behavior mimics a typical wet dog dry-off routine, without the context of being wet.
Door scratching	A bout of physical contact with the door such that continual touching was considered a single bout and if contact was broken (neither paw touching the door) the bout was ended. When one paw fell and at the same time the other paw resumed position on the door, contact was said to be unbroken.

Behaviors were predominantly analyzed from the main camera, which gave the largest view of the room (though other channels were used as a reference when dogs were not visible from that source). Proximity to the door was analyzed using the camera that exclusively monitored door activity.

Behavioral coding was performed by one main coder (MR), who was assisted by three others. Both intra- and inter-rater reliabilities were examined using intraclass correlation coefficients (ICCs), which are suitable for data coded by raters, particularly if multiple raters have been used (Landers, 2015). Specifically, ICC (2,1) is reported which, in SPSS, indicates at Two-way Random Model of single measures for Consistency (Landers, 2015). To ensure intra-rater reliability, observers coded assigned behaviors for a minimum of 6 videos and then re-coded them on a separate occasion. Intra-rater reliability was high, with an overall ICC (single measures) = 0.986 (95% Confidence Interval = 0.970–0.994). To measure inter-rater reliability, 6 videos (23%) were coded by different observers. All ICC values were high, varying slightly depending on the behaviors being coded; e.g., for door proximity, ICC (single measures) = 0.985 (95% CI = 0.922–0.997), while for body shake and door scratching, observers agreed absolutely (ICC = 1.000).

### Attachment Style Scoring

We adapted the holistic Attachment Style definitions outlined by Wanser and Udell (2019), to evaluate the nature of the attachment style between dogs and owners (Supplementary Table S2). Two judges (MR, CW) independently evaluated all 26 videos using the criteria to assess dyads according to the following 5 categories: secure, insecure-avoidant, insecure-ambivalent, insecure-disorganized, and unclassifiable. Inter-rater agreement was 96% between the two judges (i.e., judges agreed on classification of 25/26 cases). For the discrepant case, a third judge (AS) was called upon to evaluate attachment style. Consensus was reached on the classification. The third judge also independently evaluated four additional videos and agreement on classifications was unanimous.

### Data Analysis

All statistical analyses were carried out using IBM SPSS Statistics 20 (IBM, Armonk, NY, United States). A series of normality tests (binomial and Kolmogorov–Smirnov tests) to determine whether data were normally distributed revealed that several variables required transformations in order to use parametric tests; specifically, a square root transformation was performed for door scratching frequency and a log_10_ transformation was performed for CORT and CgA concentrations resulting from a positive skew, which is typical for hormonal data (Dreschel and Granger, 2009). Raw hormonal values were examined for the presence of outliers, which we defined as equal to or greater than 3 standard deviations (SDs) above the group mean; there was a single outlier in each of dog final CORT, dog final CgA, and owner final CgA and these outliers were removed from the analyses. There were no outliers in owner CORT measures. For meaningful interpretation, CORT and CgA means were back-transformed (which precludes reporting of standard error).

To account for possible effects of owner-reported separation anxiety (SA), mixed GLM ANOVAs using SA present/absent as the between-subjects factor were carried out on comparisons between how much time the dog spent with the owner versus the stranger, and initial versus final salivary analytes (within subject factors). Other analyses comparing individuals (e.g., sex comparisons) were performed using Independent Samples *t*-tests. Partial correlations between all dog and owner salivary analytes controlled for owner-reported SA. Stepwise linear multiple regression models were used to examine the effect of the following variables as predictors of SST behaviors: owner-reported SA, mean dog CORT, mean dog CgA, mean owner CORT, mean owner CgA. Given the exploratory nature of the relationships between variables in this study (e.g., owner salivary analytes and dog SST behaviors), we focused more on minimizing the reporting of Type II errors (failing to report true effects) rather than Type 1 errors (reporting false effects). Thus, statistical corrections for Type 1 errors such as the Bonferroni correction were not utilized (see Nakagawa, 2004), but we adopted a conservative alpha value of α = 0.01 (two-tailed). *P*-values ≤ 0.05 are considered marginally significant, and although reported, cautious interpretation is recommended. Further, we report the effect sizes for statistically significant *F*-values (partial eta^2^).

## Results

### Physiological Measures

#### Cortisol

##### Dogs

**FIGURE 3:**
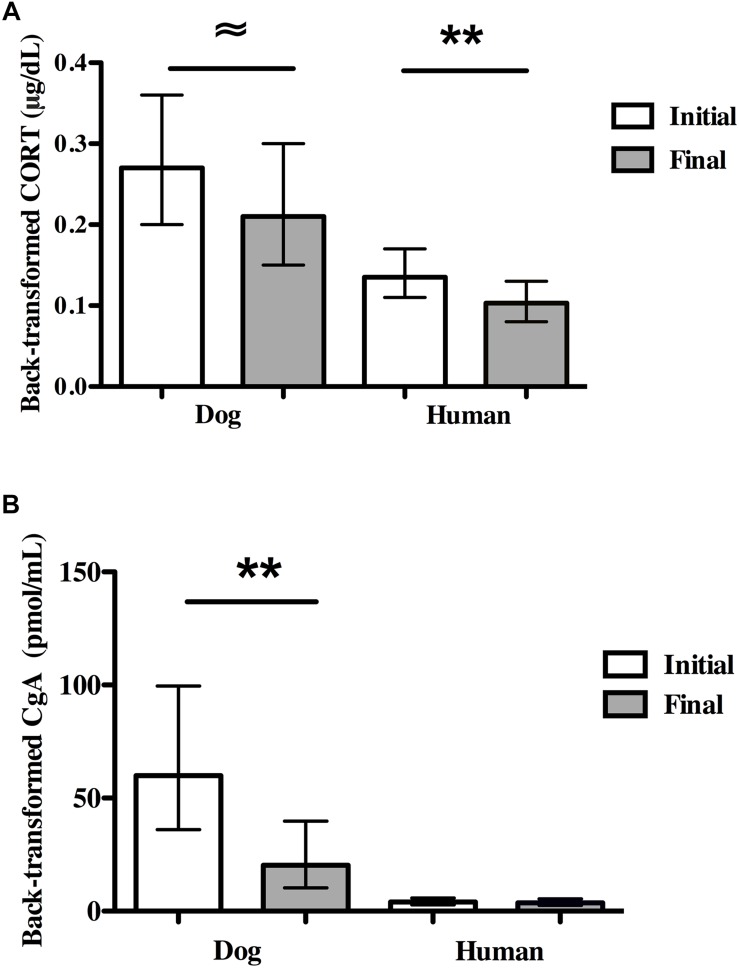
Physiological stress markers in dogs and owners in the Strange Situation Test (SST). **(A)** Initial and final salivary CORT. **(B)** Initial and final salivary CgA. Dog and human CORT concentrations decreased in the SST. In contrast, dog CgA concentrations decreased over the SST while human CgA concentrations did not change. 95% CIs are shown for the means. ∼*P* < 0.05; ^*^P < 0.01; ^∗∗^*P* < 0.001.

Average dog CORT levels decreased marginally from initial to final concentrations [*F*(1,18) = 4.45, *P* = 0.049; partial eta^2^ = 0.198; Figure 3A]. Overall, there were large individual differences in reactivity among dogs as 40% (*N* = 8) experienced an increase in CORT and 60% (*N* = 12) experienced a decrease. Dogs with owner-reported SA had higher average CORT concentrations compared to non-SA dogs (back-transformed means: 0.37 vs. 0.16 μg/dL, *F*(1,18) = 8.95, *P* = 0.008, partial eta^2^ = 0.33), and all dogs with owner-reported SA showed a decrease in CORT over the SST. There were no significant differences in initial CORT or final CORT between either sexually intact (*N* = 3) and altered dogs (*N* = 17), or male (*N* = 8) and female (*N* = 12) dogs.

##### Owners

Human CORT levels decreased significantly from initial to final concentrations (*N* = 26, *F*(1,24) = 23.55, *P* < 0.001, partial eta^2^ = 0.50; Figure 3A). There was no effect of owner-reported SA in dogs on owner CORT levels. There were no significant differences between CORT levels in men (*N* = 5) and women (*N* = 21).

#### Chromogranin A

##### Dogs

Dog CgA levels decreased significantly from the initial concentrations to the final concentrations [*N* = 18 pairs, *F*(1,15) = 10.00, *P* = 0.006, partial eta^2^ = 0.40, Figure 3B]. No dog showed an increase in CgA over the course of the SST. CgA concentrations were independent of sex of dog, the presence of separation anxiety and whether dogs were intact or desexed.

##### Owners

In contrast to dogs, there were no significant differences between owners’ initial and final CgA concentrations (*N* = 23 pairs, Figure 3B). Owner-reported SA was not related to owner CgA values. There was also no significant difference in CgA concentrations in men (*N* = 5) vs. women (*N* = 17).

#### Dog and Owner Physiological Stress

Final CORT levels for dogs were positively correlated with their owners’ final CORT levels (*r*_p_ = 0.62, *P* = 0.005, *N* = 17). Interestingly, dog initial CgA was marginally correlated with owner initial CORT (*r*_p_ = 0.58, *P* = 0.018, *N* = 14). Owner CgA levels did not correlate with either dog CORT or dog CgA.

#### Attachment Styles

All 26 dog-owner dyads were classified as having a secure attachment style. Two of these dyads were identified as “mainly securely attached with ambivalent tendencies,” and categorized as having a secure attachment style, as per the literature, e.g., (Schöberl et al., 2015; Wanser and Udell, 2019). Thus, no statistical evaluation of hormonal or behavioral data in conjunction with attachment style could be carried out, given such lack of variation.

#### Relationships Between Physiological Measures and SST Behaviors

Dogs initiated significantly higher rates of contact (contacts/sec) toward owners in both re-uniting episodes than toward strangers in the preceding episodes (Episode 4 vs. Episode 3: mean ± SE, 0.023 ± 0.004 vs. 0.008 ± 0.002, *F*(1,24) = 9.83, *P* = 0.004, partial eta^2^ = 0.29; Episode 7 vs. Episode 6: 0.028 ± 0.006 vs. 0.004 ± 0.001, *F*(1,24) = 13.08, *P* = 0.001, partial eta^2^ = 0.35). Dogs with lower CgA levels initiated more contact with their owners in both re-uniting episodes (Episode 4, β = −0.42; Episode 7, β = −0.66), while owners receiving more contact had higher CgA levels than other owners (Episode 4, β = 0.76, Episode 7, β = 0.47; overall analyses, Episode 4, *R*^2^ = 0.71; adjusted *R*^2^ = 0.66, *F*(2,12) = 14.52, *P* = 0.001; Episode 7, *R*^2^ = 0.62; adjusted *R*^2^ = 0.56, *F*(2,12) = 9.84, *P* = 0.003). Owner-reported SA was not related to differential dog-initiated contact rates or the physiological correlates.

**FIGURE 4:**
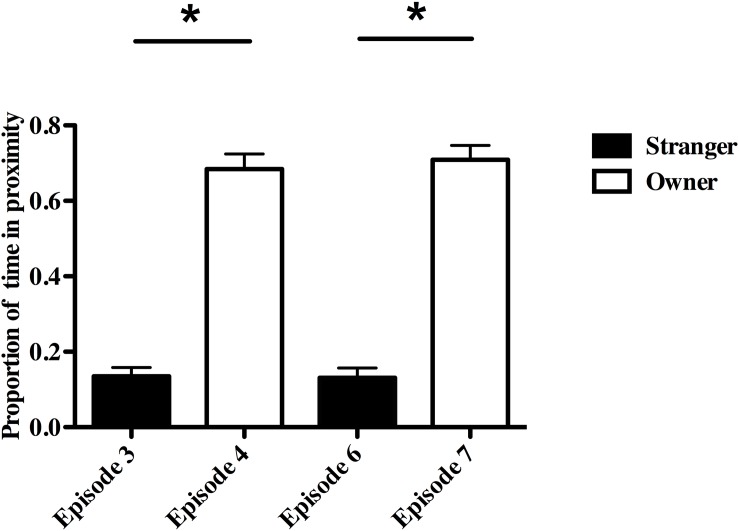
Proportion of time in close proximity to owner vs. stranger during the Strange Situation Test (SST). ^*^P < 0.01.

Dogs and owners also spent a significantly higher proportion of time near each other in the two re-uniting episodes than did dogs and strangers in the preceding episodes (Episode 4-Owner vs. Episode 3-Stranger, *F*(1,24) = 180.31, *P* ≤ 0.001, partial eta^2^ = 0.88; Episode 7-Owner vs. Episode 6-Stranger, *F*(1,24) = 195.3, *P* ≤ 0.001, partial eta^2^ = 0.89; Figure 4). This dog-owner proximity measure was related to owner, but not dog, physiology and whether the owner reported the dog to have SA (Episode 4, *R*^2^ = 0.52; adjusted *R*^2^ = 0.43, *F*(2,12) = 6.37, *P* = 0.01; Episode 7, *R*^2^ = 0.52; adjusted *R*^2^ = 0.44, *F*(2,12) = 6.52, *P* = 0.01). Owners and dogs with SA spent more time near each other in both re-uniting episodes (Episode 4, β = 0.49; Episode 7, β = 0.46) compared to other owners and non-SA dogs. Dogs and owners spent more time in proximity when owners had lower CgA in Episode 4 (β = −0.63) and higher CORT in Episode 7 (β = 0.50).

Dog salivary analytes were significantly related to the proportion of time that the dog spent near the stranger in both episodes in which the dog and the stranger were alone together. Dogs with higher CORT spent more time near the stranger in both Episode 3 (β = 0.69) and Episode 6 (β = 0.67, overall analyses, Episode 3, *R*^2^ = 0.82; adjusted *R*^2^ = 0.79, *F*(2,12) = 27.28, *P* < 0.001; Episode 6, *R*^2^ = 0.45; adjusted *R*^2^ = 0.41, *F*(1,13) = 10.67, *P* = 0.006). Dogs with high CgA levels spent less time with the stranger than dogs with lower CgA in Episode 3 (β = −0.72).

Dogs spent a significantly higher proportion of time near the door in episodes with the stranger than in episodes with the owner (Episode 3- Stranger vs. Episode 4- Owner: mean ± SE, 0.187 ± 0.025 vs. 0.055 ± 0.013, *F*(1,24) = 27.42, *P* ≤ 0.001, partial eta^2^ = 0.53; Episode 6- Stranger vs. Episode 7-Owner: mean ± SE, 0.154 ± 0.032 vs. 0.050 ± 0.011, *F*(1,24) = 10.99, *P* = 0.003, partial eta^2^ = 0.31). Two other behaviors that appeared to be responses to the SST were the body shake off seen in 21/26 dogs (81%) and door scratching seen in 13/26 dogs (50%). These behaviors were primarily observed when the dog was alone, with the stranger or just after being re-united with the owner and they were not related to SA or to any physiological measure.

## Discussion

### Preference

In this Strange Situation Test, dogs showed a distinct preference for their owners compared to the stranger as indicated by the greater proportion of time spent near owners and by the greater rate of contact initiated by the dogs toward owners, compared to strangers. As well, when left alone with the stranger, dogs spent more time near the door than they did when the owner was present. These relative preferences for the owner compared to the stranger were not influenced by whether or not the owner reported the dog to have separation anxiety. Owner preference has been a common finding in the dog-amended Strange Situation literature (Topál et al., 1998, 2005; Gácsi et al., 2001; Prato-Previde et al., 2003; Palestrini et al., 2005; Fallani et al., 2007; Palmer and Custance, 2008; Mariti et al., 2013; Rehn et al., 2013, 2014). Preference is usually defined as the degree of contact seeking and maintenance, gaze orientation, searching behaviors (e.g., waiting by the door after the owner exited) and the relative occurrence of passive (e.g., laying down) and play behaviors in the presence of the owner versus the stranger. It should be noted that there was more variation in how owners interacted with their dogs than in how the strangers interacted with them; i.e., stranger behavior was more “scripted” and involved taking a saliva sample from the dog in Episodes 3 and 6. Compared to Episode 3, the rate of dog-initiated contact with the stranger in Episode 6 was cut in half, possibly suggesting that the dog found the saliva sampling somewhat aversive and therefore avoided the stranger more on their second meeting.

### Comparison of the CORT and CgA Systems

Dogs showed a significant decrease in CgA over the SST while there was only a marginal decrease in dog salivary CORT concentrations. The differences between CORT and CgA responses in dogs may be partially explained by the biological stress systems themselves. The process of coming to a novel environment (i.e., campus) may have actually been more “stressful” or “arousing” than the SST itself, as shown through the high initial CgA levels for dogs, suggesting that the sympathetic adrenomedullary system (SAM; the faster stress system) was activated. The decrease in CgA observed during the test was probably a result of the protocol (e.g., owner returns during the fourth and the last episode) and the speed at which the SAM system changes when stress is increased and reduced (Schommer et al., 2003). The rapid decline in CgA may protect against the deleterious effects of maintaining high levels of SAM activation (Glaser and Glaser-Kiecolt, 2005).

The findings for CgA are in contrast to the slower HPA stress system for which a CORT decrease was observed in only 60% of dogs, including the ones with SA, while it increased for the rest. This variation among dogs is probably not due to the time interval we used as other studies have shown that salivary cortisol changes can be detected in dogs within this time frame (e.g., Mongillo et al., 2013; Ottenheimer Carrier et al., 2013; Schöberl et al., 2015). The increase in CORT for some dogs may have been due to the procedure being more stressful for those individuals, which, in theory could be related to factors such as the dog and/or owner personality, or the attachment style of the dyad. Indeed, a recent study showed that dogs that showed a decrease in CORT over the SST were more likely to be classified as having a secure attachment with the owner (Schöberl et al., 2015). However, in the present study all of our dog-owner dyads were classified as securely attached; therefore, none of the variation in either the hormonal or behavioral results in the current study can be related to attachment styles.

The owners’ CORT levels decreased over the SST but owner CgA levels were stable, a pattern opposite to that seen in dogs. The decrease in CORT for the owners is similar to other human testing situations (e.g., Storey et al., 2000). The owners’ low and stable CgA levels may reflect that, while the procedure was novel, it did not activate the sympathetic nervous system response as it did in their dogs.

### Influence of Owner-Reported Separation Anxiety

Some owners in this study (8/29, 28%) reported that their dogs had separation anxiety (SA), also known as separation-related disorder (Karagiannis et al., 2015), a percentage similar to that reported in a dog-owner separation and greeting study (33%, Konok et al., 2011). Owners appeared to be accurate in their assessment of SA, as these dogs showed distinct behavioral (Konok et al., 2011, the current study) and physiological (current study) differences from non-SA dogs. Dogs described by owners as having SA in this study had average CORT concentrations that were approximately twice that of dogs without SA and all of these dogs showed a decrease in CORT levels over the test period. Owner-reported SA did not influence owners’ CORT or CgA levels; thus, the higher CORT in owner-reported SA dogs was not likely due to their owners experiencing higher physiological stress. Although based on a small sample size of dogs, these findings suggest that dogs that owners believe to have separation anxiety may, in fact, have elevated CORT responses to novel or challenging situations.

Behaviorally, dogs with owner-reported SA were distinguishable in the SST only in the increased proportion of time that dogs and owners spent near each other during the two episodes (Episodes 4 and 7) when the owner and dog were alone together. As owner-reported SA was not related to rates of dog-initiated contact with the owner during these episodes, it is possible that the relationship of owner-reported SA to owner-dog proximity was mainly due to owner-initiated behaviors. Indeed, it was the owner’s physiological measures, and not the dogs’ measures in these episodes, that also predicted owner-dog proximity durations. Thus, one possibility is that the owner’s belief that the dog has separation anxiety influenced the amount of time the owner spent near the dog, or the degree to which the owner encouraged the dog to be close to him/her during these episodes of the SST.

Unlike this study, one previous study found no differences between dogs with and without separation anxiety in the time spent near or in contact with the owner during an extended version of the SST (Parthasarathy and Crowell-Davis, 2006). Similarly, no difference in “greeting affection,” a composite behavioral measure involving proximity to the owner, was found between dogs with and without SA by Konok et al. (2011), although they reported greater separation distress and greeting activity in SA dogs during separation and at reunion. These differing results raise questions regarding the extent to which “hyper-attachment” in dogs is a cause of SA, as has been suggested by some clinicians (e.g.,Sherman, 2008). Nevertheless, further consideration should be given to how owner behavior affects the behavior of dogs with SA, particularly in unfamiliar situations.

### Physiological Synchrony Between Owners and Dogs

Controlling for the possible effects of owner-reported separation anxiety in the dogs, final CORT levels in dogs were positively correlated with their owners’ final CORT levels. As well, dog initial CgA levels were positively correlated with owner initial CORT concentrations, and both owner and dog CgA predicted dog-initiated contact. It is possible that owner stress and higher initial HPA activity influenced dog SAM (CgA) activity; given the different time courses of the two stress systems, the reverse is unlikely. Owners’ CORT levels may have influenced dog physiological stress responses, measured by CORT and CgA, since, particularly in a novel setting, dogs may seek information from their owners to better understand their environment. Hormonal synchronization between dogs and owners has been recently demonstrated for dogs and handlers who participated in an agility competition; change in handlers’ CORT predicted change in dogs’ CORT levels (Buttner et al., 2015). This result was unrelated to post-competition behavioral interactions, or other variables that were investigated, suggesting that the hormonal states of owners influenced those of their dogs, and not vice versa. A possible mechanism for such findings are chemosignals released during stressful events, as seen in a recent study examining dog preference for shirts containing their owners’ sweat samples (D’Aniello et al., 2018). Dogs spent more time sniffing shirts that owners wore during a fearful situation than shirts worn when the owner was happy or when the dogs were exposed to unworn shirts (D’Aniello et al., 2018). Certainly, the effective role of medical detection dogs to alert diabetic owners to glycemic changes suggests that odor cues from humans, as well as behavioral changes, can be detected by and change the behavior of their dogs (Rooney et al., 2013; Gadbois and Reeve, 2014). It is also possible that owners responded physiologically to the perceived stress levels of their dogs; i.e., synchrony between owner and dog CORT could be due, in part, to the owner’s response to the dog’s stress behavior. As well, in the current study, we cannot rule out the possibilities that both dogs and owners may have reacted to the novel test setting independently with responses mediated by different stress systems, perhaps due to environmental/social novelty and/or anticipatory effects.

### Attachment in Relation to Physiological and Behavioral Stress

Dog CgA, in conjunction with owner CgA, was also related to dog-initiated contact toward the owner. Dogs with lower CgA and dogs whose owners had higher CgA initiated more physical contact in both reunion episodes. Although owner CgA levels were low throughout the SST, it is possible that dogs detected their owners’ low SAM arousal, and responded with increased interaction. As described above, dogs likely are able to “detect” human stress via behavior and/or chemosignals and thereby may adjust their own behaviors accordingly. In particular, the relationship between owner CgA levels and dog-initiated contact suggests that the dogs may be responding either directly or indirectly to their owner’s CgA levels, and/or to stress-related behavioral changes, an area that demands further investigation.

In contrast, the more general measure of owner and dog proximity, which could be initiated by either the dog or the owner, was related to only owner, and not dog, physiological measures. Higher proportions of time spent near each other were observed when owners had lower CgA levels (first reuniting episode) and higher CORT levels (final episode of test). This suggests that patterns of owner arousal were related not only to dog-initiated behaviors as described above, but also to owner-initiated proximity behavior. When owners had lower (SAM) arousal, the proportion of time that owners and dogs spent near each other increased (at first reunion), likely by the owner’s behavior. However, the proportion of time spent in proximity also increased at the end of the SST for owners with relatively higher HPA arousal.

Dogs with higher CORT (Episodes 3 and 6) and lower CgA (Episode 3) spent more time near the strangers than other dogs. It is important to note that during the episodes in which strangers and dogs were alone together, strangers were instructed to initiate interaction and play with the dog, and most instances of proximity would be the result of the stranger moving toward the dog. Indeed, dog-initiated contact rates toward strangers were significantly lower than they were toward owners. However, more aroused/stressed dogs (high CORT) who were not overly fearful (i.e., low SAM activation/low CgA) may have been near the stranger more often either due to increased locomotory behavior (frequently bringing the dog within proximity to the stranger) or as a means of seeking comfort (without necessarily initiating contact with the stranger).

Shake off and door scratching duration or type was not related to either CORT or CgA levels in dogs or owners. Shake off occurred in most dogs, usually just after the dog was with the stranger or just after the stranger episode ended and the owner was reunited with their dog. Therefore, shake off may be used as a way to communicate arousal or alleviate emotional tension. Shake off may also serve as a displacement behavior, which is when a behavior is used outside of its normal context because of increased arousal/stress (Wascher et al., 2010). Glenk et al. (2013) suggest that shake off may serve as a coping mechanism to manage stress, rather than serving as a manifestation of stress, a suggestion that is consistent with our results.

### Measuring CgA

Salivary CgA has almost exclusively been measured in humans (e.g., Kanamaru et al., 2006; Koh and Koh, 2007). The exception is a report by Kanai et al. (2008) where levels on research dogs were found to be much higher (3.05–3.28 pmol/mg or 3050–3280 pmol/mL) than in the current study (range 1.67 to 166.65 pmol/mL). It is difficult to determine whether this difference is due to assay characteristics or instead, reflects the fact that the stress levels of dogs with close attachments to their owners, even in this novel situation, are lower than those of research dogs. The high levels of initial CgA in dogs, and the positive relationship between dog CgA levels and the time the dog spent near the door in the first two episodes of the SST (with owner present), suggests that general distress or arousal may be reflected by CgA measures. CgA holds significant promise as a biomarker of sympathetic nervous system activity in dogs (Stridsberg et al., 2014) and further understanding of salivary CgA as an indicator of stress will enhance dog welfare-related research. However, we urge cautious interpretation of the current results for salivary CgA, as the time course of CgA is poorly understood and it is possible that our sampling intervals did not capture the entire effect.

## Conclusion

Dog-owner attachment, as measured by the SST, was reflected by changes in dog and owner physiological stress measures, and dog behavior. Similar to previous studies, dogs showed a preference for owners compared to strangers in the SST. Physiological stress responses of both the dog and the owner are related to variation in proximity-seeking behaviors during the SST. Dog CORT, a measure of HPA axis activation, and dog CgA, an indicator of SAM activation, correlated with owner CORT values in the SST, indicating possible hormonal synchrony in the dyad. Future research should attempt to examine hormonal synchrony within dyads to eliminate alternate explanations such as similar independent reactions to novelty/anticipation. Since this study is the first to use salivary CgA as a measure of stress responses to a behavioral/psychosocial challenge in dogs, additional work on the activity of CgA in such a context is required. As well, although the sample size for dogs with owner-reported separation anxiety was small, these results further support findings that aspects of the owner-dog attachment relationship likely exert influences on dog and owner interactions that may be reflected in physiological responses to stressors.

## Data Availability

The datasets generated for this study are available on request to the corresponding author.

## Ethics Statement

This study was carried out in accordance with both the recommendations of the Tri-Council Policy Statement on Ethical Conduct for Research Involving Humans (T2) and the Canadian Council on Animal Care (CCAC). All human subjects gave written informed consent in accordance with the Declaration of Helsinki. The protocol was approved by both the Interdisciplinary Committee on Ethics in Human Research (ICEHR, protocol #2012-320-SC) and the Institutional Animal Care Committee (IACC protocol #12-01-CW) at Memorial University of Newfoundland.

## Author Contributions

MR, CW, and AS conceived the idea, designed the study, and developed the methods, with significant inputs from RA. MR performed the study. MR and CW analyzed the data. MR, CW, and AS wrote the manuscript. CW and AS contributed to the resources.

## Conflict of Interest Statement

The authors declare that the research was conducted in the absence of any commercial or financial relationships that could be construed as a potential conflict of interest.

## Footnotes

1 http://play.psych.mun.ca/_apps/log/
