# Supplementary material for: Physiological Indicators of Attachment in Domestic Dogs (Canis familiaris) and Their Owners in the Strange Situation Test
Source: Front Behav Neurosci. 2019 Jul 23;13:162. doi: 10.3389/fnbeh.2019.00162 (PMC6664005; doi:10.3389/fnbeh.2019.00162)
Supplement: Supplementary file 1 [file Table_1.docx]

**S1 Table. Dog Breed Information.** The number of dogs from each breed is indicated in parentheses.

| Breed | Mass (kg) | Height (cm) |
| --- | --- | --- |
| Beagle (5) | 10-11 | 33-38 |
| Cavalier King Charles Spaniel (1) | 5.5-8 | 30-33 |
| Collie (1) | 22.5-34 | 56-66 |
| Eurasier (1) | 31-32 | 60 |
| German Sheppard (1) | 34-36 | 64 |
| Golden Doodle (1) | 22-40 | 50-60 |
| Labrador Husky (1) | 27-45 | 53-60 |
| Labrador Retriever (3) | 27-34 | 57-62 |
| Miniature Golden Doodle (1) | 7-8 | 28-38 |
| Newfoundland (1) | 54-67.5 | 66-71 |
| Old English Bull Dog (1) | 25-36 | 40-50 |
| Pit Bull Terrier (1) | 10-35 | 35-60 |
| Samoyed (1) | 16-30 | 48-60 |
| Yorkshire Terrier (1) | 1-3 | 20-23 |
| Mixed Breed (9) | 11-36 | 30-76 |

Note: All source material for measurements are from the Canadian Kennel Club breed (2015) standard guidelines or from other internet sources (dogtime.com and dogbreedinfo.com).
