# Supplementary material for: Physiological Indicators of Attachment in Domestic Dogs (Canis familiaris) and Their Owners in the Strange Situation Test
Source: Front Behav Neurosci. 2019 Jul 23;13:162. doi: 10.3389/fnbeh.2019.00162 (PMC6664005; doi:10.3389/fnbeh.2019.00162)
Supplement: Supplementary file 2 [file Table_2.DOCX]

**Secure:** Dog **approaches owner promptly at reunion and follows**,

**makes physical contact or signals for contact**,

**seeks and is comfortable with contact**.

**Little or no gaze aversion** or **proximity avoidance**

**Little or no resistance to contact** or interaction

**Insecure:**

**A. Insecure-Avoidant:**

Dog shows **little tendency to approach**, to **seek contact**, **or to follow**.

Dog **turns or looks away during reunion**.

Dog shows **lack of response to invitations to approach or interact for 30 s** or more.

Dog **explores the room** and objects **during pre-separation and post-separation**.

There is **little active search for owner**.

**B. Insecure-Ambivalent**

On reunion, they **mixed persistent distress with efforts to maintain physical contact**

**and/or physically intrusive behavior directed toward the owner.**

A degree of **conflict regarding physical contact or play activities** (e.g., the dog wished to

maintain contact and was uncooperative with the owner’s attempt to encourage play or

exploration, or the owner maintained firm physical contact which the dog merely passively

tolerated.) ^[[1]](#footnote-1)^

**C. Insecure-Disorganized**

Evidence of **strong approach avoidance conflict** or **fear on reunion**

(e.g., circling owner, hiding from sight, rapidly dashing away on reunion,

“aimless” wandering around the room, shying away from contact, or proximity).

“**Dissociation” may be observed, i.e., staring into space** without apparent cause;

**still or frozen posture for at least 20 s** (in the non-resting, non-sleeping dog).

**Unclassifiable:**

Dogs showed **ambiguous evidence of disorganization or other disturbance**-

(e.g., “depressed”-a marked lack of enthusiasm in a dog that otherwise seemed secure or

showed other behavior suggesting a neurologic or compulsive disorder).^[[2]](#footnote-2)^

1. Dogs who the judges agreed seemed essentially secure but with ambivalent tendencies, were included in the secure group. [↑](#footnote-ref-1)
2. Unclassifiable dogs to be excluded from further analysis on dog attachment. [↑](#footnote-ref-2)
